# Editorial for the Special Issue on Micro/Nano Devices for Blood Analysis, Volume II

**DOI:** 10.3390/mi13020244

**Published:** 2022-01-31

**Authors:** Susana O. Catarino, Graça Minas, Rui Lima

**Affiliations:** 1 Center for MicroElectromechanical Systems (CMEMS-UMinho), University of Minho, 4800-058 Guimarães, Portugal; 2 LABBELS–Associate Laboratory, Braga/Guimarães, Portugal; 3 CEFT, Faculdade de Engenharia da Universidade do Porto (FEUP), Rua Roberto Frias, 4200-465 Porto, Portugal; 4 MEtRICs, Mechanical Engineering Department, Campus de Azurém, University of Minho, 4800-058 Guimarães, Portugal

The development of micro- and nanodevices for blood analysis continues to be a growing interdisciplinary subject that demands the careful integration of different research fields. Particularly, such devices target the precise integration of mechanics, microfluidics, micro/nanotechnologies, chemistry, optics, electronics, materials engineering, informatics, biotechnology and medicine, among many other fields.

Microelectromechanical systems (MEMS) combine electrical, optical and mechanical components at the microscale level and are a result of continuous efforts in the miniaturization of mechanical microdevices. The integration of these devices with microflows, in the form of micropumps, microvalves or even sensors, has gained the interest of scientific communities dealing with fluid flow and transport phenomena happening at the microscale. Microfluidic systems have considerable advantages over macroscale devices, as they offer the ability to work with low power consumption, small samples and reagent volumes, provide the good manipulation and control of samples, decrease the reaction times and allow multiplexer and parallel operations to be carried out in one single step. Thus, microdevices have great potential in developing portable and point-of-care diagnostic devices, particularly for cellular and blood analyses. Moreover, the recent progress of nanotechnology is gaining popularity and has expanded the areas of application of microfluidic devices, which include the manipulation and analysis of flows on the scale of DNA, proteins and nanoparticles, which has led to the creation to a new field—nanoflows. In spite of the enormous scientific achievements that micro and nanofluidics have had in recent decades, they are still considered to be in the early stages, with some drawbacks and challenges to overcome, such as the difficulty in achieving cost-effective, large-scale production and the complete understanding of the physics of fluids at the micro and nanoscale level. As a consequence, many research groups worldwide are devoting significant efforts to improvements of research regarding microfabrication and microfluidics to enhance the potential of such microdevices to develop portable and point-of-care diagnostic devices, particularly for blood analysis, as we target in this book.

Following the success of the Special Issue “Micro/Nano Devices for Blood Analysis”, which led to the publication of a book with the same title [1], once again, we invited the scientific community to participate in and submit their research to the Special Issue “Micro/Nano Devices for Blood Analysis, Volume II”. Researchers from different areas and backgrounds cooperated actively and submitted high-quality research, focusing on the latest advances and challenges in micro- and nanodevices for diagnostics and blood analysis, micro- and nanofluidics, technologies for flow visualization and diagnosis, biochips, organ-on-a-chip and lab-on-a-chip devices and their applications to research and industry. Thus, this book assembles ten scientific documents, including comprehensive reviews and experimental research papers, in the areas of microsystems design, simulation, experimental characterization and diagnosis.

In this Special Issue, Fadlelmoula et al. [2] presented a review of current Fourier Transform Infrared (FTIR) spectroscopy techniques and their potential for human blood analysis. The authors discussed the advancements of the technology and its variations in recent decades and reported the latest research focusing on FTIR spectroscopy applications in the biological field, which distinguish between healthy and pathological samples and help in the diagnosis of different diseases. Finally, the authors explored the current challenges of FTIR integration into microfluidic and lab-on-a-chip devices [2].

In another review, regarding the topic of blood flows and RBCs characterization and visualization, Carvalho et al. [3] carefully reviewed and compared different manual and automatic image analysis segmentation methods for blood flow studies in microfluidic domains. The authors discussed the role and importance of image analysis in the processing of blood flow data when acquired from high-speed video microscopy. While the authors showed that manual methods are reliable, they are highly time-consuming, user-intensive and repetitive, and the results can be subject to user-induced errors. For that purpose, the authors presented, discussed and compared manual and automatic methods that are able to achieve similar accuracy in the data analysis, both to individually track RBCs and measure cell-free layer thicknesses in different kinds of microchannels [3].

In addition to these reviews, the Special Issue also contains several research papers that cover different subjects related to microfluidic structures, cells characterization, data analysis and diagnosis. Hu et al. [4] presented a novel method of creating small-diameter artificial vascular grafts (with diameters below 6 mm), which can form a controllable, multi-material composite microstructure on the inner wall of a blood vessel. The authors combined different additive composite molding in the inner walls and outer walls of artificial vessels in order to promote endothelialization and ensure the stability, good structure and mechanical performance of the structure [4].

Al-aqbi et al. [5] also developed novel microfluidic devices for blood applications. The authors proposed a new geometry for blood plasma filtration based on a nano-junction formed by dielectric breakdown. The authors achieved the on-chip filtration and out-chip collection of blood plasma from a whole blood sample, with a high extraction yield of 62%, within less than 5 min. The authors characterized the filtered plasma, showing that it presented a low concentration of analytes from the whole blood, confirming the success of the proposed microdevice [5].

Adekanmbi et al. [6] focused on the design, numerical modeling and simulation of a microfluidic device for the dielectrophoretic separation of infiltrating ductal adenocarcinoma cells (ADCs) from isolated peripheral blood mononuclear cells (PBMCs). The proposed device successfully combined a 1.4 mm long, Y-shaped microfluidic channel with semi-circular insulating constrictions and dielectrophoresis promoted by a DC electric actuation with varying voltage conditions [6].

Kang [7] presented a microfluidic-based biosensor for the measurement of blood viscosity and red blood cells’ (RBCs’) sedimentation rates. The author used two air-compressed syringes to deliver blood samples and a reference fluid into a T-shaped microfluidic channel and measured the average velocity of the fluids using micro-particle image velocimetry and digital image processing. The sedimentation rate was quantified based on the analysis of the image intensity of the blood sample over time. Then, blood viscosity was determined using a parallel co-flowing method with a correction factor. The author was able to measure blood viscosity and the RBCs’ sedimentation rates consistently for samples with hematocrit 30%, 40% and 50% [7].

The same author [8] also designed a T-shaped microfluidic device with two inlets, one outlet, two guiding channels (one for the blood sample and the other for the reference fluid) and one co-flowing channel for the measurement of blood viscoelasticity through the analysis of the interface variations in the co-flowing streams of the sample and the reference fluid. For that purpose, the author proposed a sinusoidal flow-rate pattern to pump the blood samples, while the reference fluid was pumped at a constant flow rate. Based on that setup, the author measured the compliance (influenced by the period of the flow rate) and the viscosity of the fluid (dependent on the hematocrit and solvent). Finally, from those variables, the author analytically calculated and monitored the viscoelasticity of the blood samples [8].

Related to his other published works, Kang [9] also studied, under periodic on–off blood flows, the contributions of air compliance to the dynamic blood flows in microfluidic channels for the successful evaluation of the mechanical properties of blood samples. The author analyzed the image intensity and interface in a co-flowing channel and concluded that air cavities in the blood sample syringe contributed to delays in the transient behaviors of blood flows, hindering the quantification of RBCs’ aggregation and blood viscoelasticity [9].

In addition to the referred works, which focused on the mechanical characterization of blood samples and RBCs in microfluidic devices, one paper, presented by Pinho et al. [10], explored the potential of microfluidic structures for the integration of organ-on-a-chip technologies, targeting personalized cancer medicine. The authors developed preclinical models of colorectal cancer by combining microfluidic technologies with 3D, patient-derived tumor organoid models. The authors used a low-cost microfluidic device to culture and expand the colorectal cancer organoids and evaluated their viability and proliferative activity, having concluded that the developed chip had the potential to generate organotypic structures for disease modeling and drug screening applications [10].

Finally, the last paper, while not being related to microfluidics, presents a novel microdevice for malaria diagnosis based on blood analysis. Costa et al. [11] designed, simulated and experimentally characterized bandpass optical filters, based on multilayers of thin films, for implementation in a device for reflectance-based malaria diagnosis. The authors proposed the detection of malaria parasites in RBCs through the optical quantification of hemozoin. For that purpose, a set of eight thin-film optical filters, based on multilayer stacks of MgO/TiO_2_ and SiO_2_/TiO_2_ thin films, with high transmittance and low full width at half maximum at specific wavelengths in the 400 nm–800 nm range of the optical spectrum, was designed and characterized. The obtained results showed that optical measurements with the developed filters allowed one to distinguish between healthy and malaria-infected samples, up to a detection limit of 12 parasites/μL of RBCs.

As Guest Editors, we hope this book can, once again, provide an opportunity for the biomedical, biotechnology and engineering communities to access novel information and knowledge, particularly in the applications of micro and nanofluidics to biomedical fields. We believe this book can contribute significantly to the scientific community as a demonstration of the latest results, achievements, breakthroughs, challenges and trends in micro and nanodevices for diagnostics and blood analysis, cells’ characterization, micro and nanofluidics, flows visualization, MEMS, biochips, organ-on-a-chip and lab-on-a-chip devices and their application to research and industry.

As a final remark, we would like to acknowledge, congratulate and thank all of the researchers and authors for submitting and contributing their original manuscripts to this Special Issue, as well as the reviewers for their time and efforts and their careful participation in the peer-review processes, which were essential to improve the quality of the documents.
